# The Evolving Role of Nucleotide-binding Oligomerisation Domain-like Receptor Pyrin Domain 3 Inflammasome Activation in Vascular Endothelial Cells: A Review

**DOI:** 10.21315/mjms2022.29.2.2

**Published:** 2022-04-21

**Authors:** Razif ABAS, Rusliza BASIR, Suryati MOHD THANI, Safuraa SALIHAN, Azmah SAAT, Nurul Hayati MOHAMAD ZAINAL, Siti Fadziyah MOHAMAD ASRI, Nur Izah AB RAZAK, Nurul Huda MOHD NOR, Nur Aqilah KAMARUDDIN

**Affiliations:** 1 Department of Cardiovascular Sciences, University of Leicester, Glenfield Hospital, United Kingdom; 2 Department of Human Anatomy, Faculty of Medicine and Health Sciences, Universiti Putra Malaysia, Selangor, Malaysia; 3 Department of Anatomy, Faculty of Medicine, Universiti Kebangsaan Malaysia Medical Centre, Kuala Lumpur, Malaysia

**Keywords:** endothelial cells, NLRP3 inflammasome, vascular, inflammation

## Abstract

In the vascular wall, defence against pathogenic damage requires a group of monocytes, the endothelium, dendritic cells, macrophages and a subsequent involvement of pattern recognition receptors anticipating damage-associated molecular patterns (DAMPs) to initiate an innate immune response. The endothelium plays a crucial role in regulating the duration, location and extent of the inflammatory cascade to ensure a definitive immune defence. Molecular changes in the expression of chemokines and cell adhesion molecules ensure protective responses against infection and injury. The multiprotein oligomer complex nucleotide-binding oligomerisation domain (NOD)-like receptor pyrin domain 3 (NLRP3) inflammasome plays a key role in the activation of inflammatory processes in response to DAMPs and pattern-associated molecular patterns. As a result of NLRP3 inflammasome activation, caspase-1 is activated and interleukin-1β (IL-1β) is produced. Caspase-1 is the main mediator of inflammatory feedback to tissue injury, and it is engaged both in the initiation of the inflammatory response and in the induction of cell death. NLRP3 inflammasome promotes further inflammatory responses and pyroptosis in the vascular endothelium; thus, its optimum regulation is crucial in cardiovascular homeostasis. This review outlines our current perception of the role of NLRP3 in vascular endothelial cells.

## Introduction

The innate immune system engages the aggregated actions of physical barriers, plasma protein activation and a collection of immune cell types, including monocyte-macrophages, thus making a prompt response to pathogens within hours (1). Pattern recognition receptors (PRRs) are essential baseline components of the innate immune response that recognise subsequent effects through pathogen-associated molecular patterns (PAMPs), which are triggered by pathogens, and damage-associated molecular patterns (DAMPs), which are released because of ischaemia, trauma or any form of tissue/cellular destruction (2). Nucleotide-binding oligomerisation domain (NOD)-like receptors (NLR), toll-like receptors (TLR) and absent in melanoma 2-like receptors (ALR) are also involved in PRR structure formation. ALR and NLR sense signals from pathogens intracellularly in the cytoplasm, whereas TLR senses extracellularly on the cell surface. Both ALR and NLR trigger nuclear factor-kappa-light-chain-enhancer of activated B cells (NF-κB) for further inflammasome protein component upregulation.

Inflammasomes are intracellular multiplex protein complexes that have been extensively studied over the past 20 years (3). A mass of inflammasome is formed in response to unwanted PAMP and DAMP senses, thus producing Signal 1 or the priming process. A subsequent Signal 2 or the activation process, is then constructed for the final biological activity of the inflammasome through the maturation of interleukin-1β (IL-1β) and interleukin-18 (IL-18) end-stage productions. Interestingly, inflammasome activation also results in the production of pyroptosis, which is pro-inflammatory programmed cell death.

**Figure 1:**
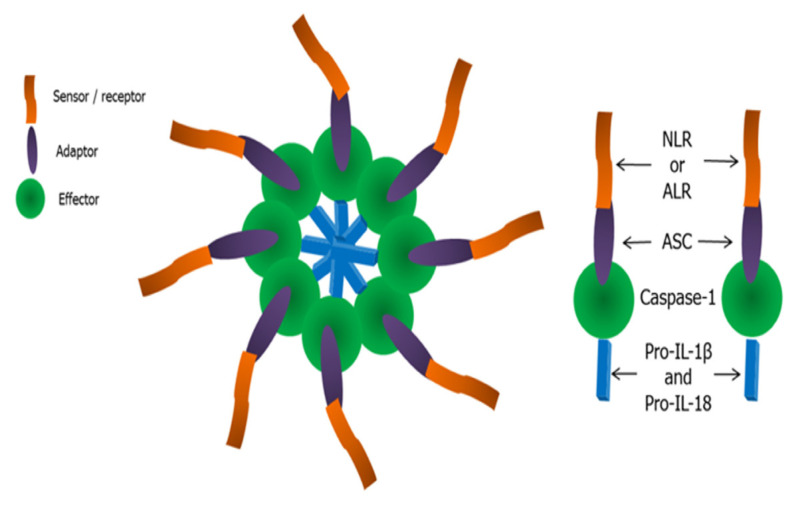
The multiplex protein of an inflammasome. The monomeric basic structure of an inflammasome comprises an adaptor, a sensor protein and an effector, forming the multiplex protein complexes of the NLRP3 inflammasome. Pro-IL-1β and pro-IL-18, both pro-inflammatory cytokines, are immature

The essential organisation of the inflammasome multiplex protein comprises three units: i) a downstream adaptor; ii) an upstream receptor and iii) a protein effector. An apoptotic speck-like protein containing the caspase-1 activation and recruitment domain (CARD) (apoptosis-associated speck-like protein containing a CARD [ASC]) comprises the adaptor. NLR or ALR establishes receptor formation, while pro-caspase-1 completes the formation of the effector molecule (4) (Figure 1). Subsequently, the maturation of caspase-1 cleaves the immature inflammatory cytokines pro-IL-1β and pro-IL-18 to mature IL-1β and IL-18, respectively, thus initiating the inflammatory defence cascade.

There are 22 types of NLR proteins, but NLRP1, 3, 6, 7, 12 and NLRC4 are known to comprise the inflammasome multiplex protein (5). ALR and retinoic acid-inducible gene 1-like receptors, which are non-NLR proteins, are also associated with inflammasome multiplex protein production (6).

Inflammasome complexes are essential for hosts to defend against foreign bodies, PAMPs or DAMPs. The insufficient production of the inflammasome leads to the massive production of pro-inflammatory cytokines, thus promoting oncogenic, metabolic, autoimmune and autoinflammatory effects (7). However, the exact mechanisms of its optimum function remain doubtful and questionable. Therefore, in this review, we elaborate on the mechanisms of inflammasome production, especially in the NLRP3 inflammasome, from different triggers. The progression of inflammatory diseases, such as atherosclerosis, is beneficially interrupted by the early detection of pathogenic agents and their inflammasome pathways.

## Discussion

### NOD-like Receptor Pyrin Domain 3 Inflammasome

The NLRP3 inflammasome is the most extensively investigated among all the NLR proteins. It acts against diverse types of pathogens, such as bacterial, fungi and viruses. The alteration of NLRP3 genetic coding of cryopyrin protein manifests at a higher number of active IL-1β production, which leads to familial cold autoinflammatory syndrome, Muckle–Well syndrome and neonatal-onset multisystem inflammatory disease (6). NLRs are protein receptor sensors within the cytosolic compartment, and they are associated with the activation of the immune response during pyroptosis (8). Pyroptosis is a diverged form of apoptosis in that programmed cell death in pyroptosis requires inflammatory feedback. Pyroptosis is manifested by cell swelling, membrane burst and pore development, thus promoting the discharge of inflammatory cytokines.

NLR has three main domains: i) C-terminal leucine-rich repeat (LRR) domain; ii) central domain and iii) the most crucial N-terminal domain (9). An LRR domain forms the C-terminal repeat domain, while nucleotide-binding and oligomerisation domains (neuronal apoptosis inhibitor protein {NAIP}, MHC class 2 transcription activator {C2TA}, incompatibility locus protein from Podospora anserina {HET-E} and telomerase-associated protein {TP1} [NACHT]) comprise the central unit. Both LRR and NACHT are known to negatively feedback the NLR sensor in the non-appearance of any triggers (10). The baculoviral inhibitor apoptosis repeat domain, pyrin domain and CARD also establish the N-terminal domain. As a result, the NLRP3 inflammasome is formed through the assembly of a NOD-like receptor protein containing the pyrin domain 3 (11).

**Figure 2:**
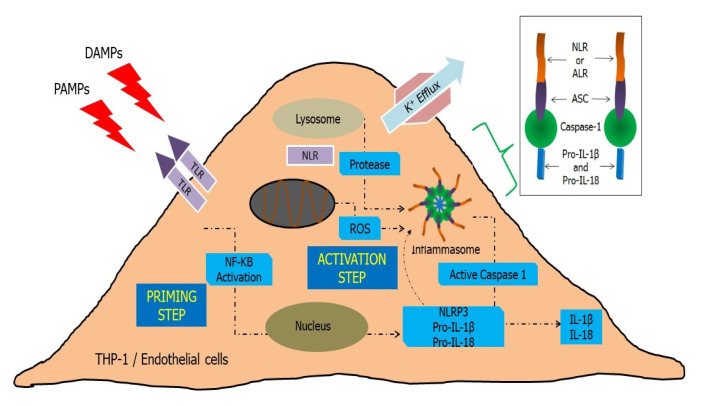
Schematic diagram showing endothelial cell NLRP3 inflammasome activation through the priming and activation steps. The priming step promotes NF-kB production, stimulating the activation steps to produce active IL-1β and IL-18, which are the NLRP3 inflammasome end products

NLRP3 is expressed as dormant in its monomeric structure. It requires the coupling of an effector protein (caspase) with CARD and an adaptor protein (ASC) with CARD to form an oligomerised architecture (CARD–CARD). The CARD domains carry protein monomers of pro-caspase-1 into adjacency, thus forming a multiplex protein structure called the NLRP3 inflammasome complex (12). Consequently, this complex triggers the self-cleavage of pro-caspase-1 to form active caspase-1. This mature form of caspase-1 initiates the proteolytic cleavage of immature pro-IL-1β and pro-IL-18 to generate active IL-1β and IL-18, respectively, which are the end products of the NLRP3 inflammasome, positively feedbacking the immune cascade response (13) (Figure 2).

Numerous concepts have been proposed for NLRP3 inflammasome complex activation. Pore formation by bacterial toxin leads to extracellular adenosine triphosphate (ATP) accumulation through the P2X7 receptor, which activates potassium efflux (14). Moreover, phagosomal and burst vesicles are formed by phagocytotic PAMPs and DAMPs, which release the reactive oxygen species (ROS), lose their phagosomal acidity and discharge the lysosomal calcium and cathepsin B, thus activating the NLRP3 inflammasome formation (15).

To date, the definite mechanism and cascade of coordinating NLRP3 inflammasome production remain ambiguous, but as with all inflammasomes, first and second signals are required (16). A typical priming signal of the NLRP3 inflammasome activates an immature IL-1β gene expression precursor of NLRP3 through the NF-κB transcription factor. Later, the inflammasome component is assembled in a multimeric form through the stimulation of the activation signal (17). Three proposed active processes (18) are expected: i) amplification of NLRP3 activity by protein ‘X’ production through the mitochondrial ROS triggering of a pore-forming toxin; ii) active caspase-1 phagocyte particulate matter initiating lysosomal or endosomal damage and iii) unclear mechanism of increased ATP and calcium extracellularly that may relate to excessive potassium efflux. These processes activate the NLRP3 inflammasome development.

A study (19) showed that PAMPs, including a microbe-derived lipopolysaccharide, are potent priming signal agents, while PAMPs and DAMPs play a main role in subsequent activation signals. Any toxin from pathogens, including nigericin (*Streptomyces hygroscopicus*), maitoxin (*Marina dinoflagellates*), aerolysin (*Aeromonas hydrophila*), cholera toxin B (*Bacillus brevis*) and α-toxin (*Staphylococcus aureus*), are an example of PAMPs. Asbestos, cholesterol crystals, uric acid, alum, crystals, silica, ATP and hyaluronan are considered DAMPs that activate the priming of NLRP3 inflammasome (20). Interestingly, the literature has also emphasised that the sole priming signals of NF-κB activation are insufficient to activate the production of the NLRP3 inflammasome protein complex; thus, a combination of priming and activation signals is required (21).

### The Importance of NOD-like Receptor Pyrin Domain 3 Inflammasome in Endothelial Cells

Since the early 1980s, the integrity and functions of endothelial cells, either in vivo or in vitro, have been widely investigated by many researchers but not to the extent of inflammasome connections (22). Although the involvement of macrophages in NLRP3 inflammasome investigations has been extensively studied, a paucity of research on NLRP3 inflammasome involvement in endothelial cells has been observed. Here, we review the current perception of the evolving role of endothelial cell function and NLRP3 inflammasome activation.

Endothelial function deficiency may prompt ongoing inflammation, tissue swelling and the development of thrombi. These progressions ultimately lead to endothelial dysfunction. During the progression from innate to adaptive and from acute to chronic inflammation, the dysmorphology of endothelial cells appears, with a quick aggregation of neutrophils and an extensive vascular leakage of the plasma proteins. Innate immune cells are important in PRR-related innate responses, but the effect of nonimmune cells (e.g. endothelial cells) is also critical. Subsequently, IL-1β, a pro-inflammatory cytokine, is expressed during the endothelial reaction (23). The initiation of NLRP3 inflammasome in endothelial cells under pathophysiological conditions may exacerbate endothelial weakness, prompting hypertension, obesity, diabetes, neuroinflammation, retinopathy, atherosclerosis, cerebrovascular accident and malignancy.

### NOD-like Receptor Pyrin Domain 3 Inflammasome and Cardiovascular Homeostasis

Human umbilical vein endothelial cells (HUVECs) and human aortic endothelial cells (HAECs) express NOD1, which is one of the NLRP3 inflammasome subunits. NF-κB activation triggered by inflammatory mediator exposure potentiates a lower basal NOD2 expression (22). NOD1 and NOD2, which are both peptidoglycan fragments, can distinguish the degradation products of bacteria: gram-negative bacteria predominantly for NOD1 and gram-positive for NOD2. Consequently, cell cytosol endocytoses these bacterial products through the peptide transporters PepT1 and PepT2 (24).

Dysfunctional vascular endothelial cells, with their defence mechanisms, play an important role in atherosclerosis development. The NLRP3 inflammasome can be produced by cholesterol crystals that later induce macrophage activation. The sub-endothelial accumulations of T-cells, lipids, cholesterol and principally macrophage foam cells form an initial lesion of atherosclerosis evidenced by the presence of a fatty streak (25). In a murine atherosclerosis model, a 30% reduction in atherosclerotic plaques was shown in the case of IL-1β gene deletion (26).

In studies involving the plasma of patients with peripheral arterial diseases, the stimuli of cholesterol crystals in the HAEC can activate the intracellular NLR receptors of endothelium signals in the macrophages and trigger caspase-1 activation through cellular response. As a result, pro-IL-1β is cleaved by active caspase-1 to form an active IL-1β, an influential pro-inflammatory cytokine that results in atheroma formation. However, this model demonstrates a lesser activation of NLRP3 compared with a higher NLRP1 expression (27). An increase in NLRP3 inflammasome formation in endothelial cells is also activated by increased integrin α5 in lipid rafts due to the presence of oxidised low-density lipoprotein and oscillatory shear stress, contributing to the development of atherosclerosis (28).

In addition to arterial blood flow disturbance, which causes oscillatory shear stress in vascular endothelial cells, lipopolysaccharide and cholesterol crystals also induce vascular damage through the activation of sterol regulatory element binding protein 2 (SREBP2) (29). The synthesis of cholesterol formation is required by SREBP2 as a key regulator. SREBP2 activation simultaneously stimulates NLRP3 transcription and nicotinamide adenine dinucleotide phosphate (NADPH) oxidase 2, with a short-lived ROS elevation, which subsequently activates the formation of NLRP3 inflammasome. In this case, note that the active IL-1β end product initiates endothelium dysfunction and atherosclerosis through the production of intercellular adhesion molecule-1 (ICAM-1), vascular cell adhesion molecule 1 (VCAM-1), E-selectin and monocyte chemoattractant protein 1, promoting monocyte recruitment and foam cell formation (30).

### NOD-like Receptor Pyrin Domain 3 Inflammasome-associated Activation Agents

Studies on *Lactobacillus casei*-inducing coronary arteritis in a mouse vascular endothelial cell model in Kawasaki disease revealed the release of cathepsin B, which is a lysosomal protease. This product accelerates NLRP3 inflammasome formation by increasing lysosomal membrane permeability and releasing cytosolic lysosomal cathepsin B. Furthermore, immunohistochemistry showed an increased expression of endothelial–leukocyte adhesion and VCAM-1 in the coronary arterial endothelium, correlating with NLRP3 inflammasome enhancement (31).

Cadmium (Cd) has also been demonstrated to accumulate intracellular ROS, which induces NLRP3 inflammasome activation in HUVECs. Cd has been proven to activate active IL-1β production through caspase-1 cleavage. It promotes pyroptosis in HUVECs and it is mediated by mitochondrial ROS generation. Xenobiotic pregnane X receptor (PXR) agonists and ATP were also shown to activate NLRP3 inflammasome production in endothelial cells. NLRP3 inflammasome promotes oxidised low-density lipoprotein release, thus activating cholesterol crystals, which will lead to cardiovascular disease and atherosclerosis (32).

The release of high mobility group box 1 protein (HMGB1) plays a role in the inflammasome signalling pathway in endothelial cells. HMGB1 promotes the pro-inflammatory ion-promoting activity of cytokines through the processes of DNA transcription, repair and replication (33). Caspase-1 activation also triggers the release of fibroblast growth factor. HMGB1 and fibroblast growth factor prime the inflammasome cascade through NF-κB activation, releasing the pro-inflammatory cytokines TNF-α and pro-IL-1β (34). Studies on lung endothelial cells have shown endothelial NADPH oxidase activation in the case of haemorrhagic shock or acute lung injuries, triggering respiratory NLRP3 inflammasome production. NLRP3 inflammasome production is also triggered by ROS production in the lungs through polymorphonuclear neutrophil NADPH oxidation (35).

Lipopolysaccharide-treated monocytic cell microparticles are believed to activate NF-κB in endothelial cell inflammasome pathways, such as NF-κB and extracellular signal-regulated kinases (ERK) 1 and 2, by expressing P-selectin glycoprotein ligand-1 and lymphocyte function-associated antigen-1 to produce inflammasomes. Microparticles are waste products from the degradation of cell death or during cell activation (36). They induce the production of different types of adhesion molecules (e.g. E-selectin and ICAM-1), which facilitate the binding of white blood cells to the endothelium to activate the adaptive immune system. The inflammasome production is facilitated by ATP activation on the cell surface through the P2X7 receptor, forming pores that lead to IL-1β synthesis release (37).

The thioredoxin interacting protein (TXNIP) is expressed by disrupted blood flow in constricted vascular regions, especially in the aortic arch and its terminal branches (38). TXNIP is a potent anti-inflammatory transcription factor in endothelial cells belonging to the α-arrestin family, acting as an inhibitor of Kruppel-like factor 2 (KLF2) expression. Thus, KLF2 promotes the upstream regulation of NLRP3 protein transcription and cell adhesion molecule expression. Conversely, evidence shows that steady blood flow activates the mitogen-activated protein kinase (MEK)5-ERK5 pathways, protein kinases that belong to the mitogen-activated protein kinase (MAPK) family, thus increasing KLF2 expression (39). Aside from these scenarios, endothelial dysfunction in uremic or chronic kidney disease patients is initiated by hyperphosphatemia. The overexpression of TLR4 and NLRP3 inflammasome components through NF-κB is triggered by serum uremic, which acts as DAMPs to promote endothelial cell dysfunction (40).

Cerebral malaria infection by *Plasmodium falciparum* produces erythrocyte adhesion in brain capillary endothelial cells because of excessive inflammatory cytokine secretion, promoting NLRP3 inflammasome activation. A heme polymer hemozoin is formed by the degradation product of *P. falciparum* after the polymerisation of haemoglobin, thus worsening the inflammatory process. *S. aureus* is also believed to activate α-haemolysin toxin to trigger cysteine proteinase enzymes, which cleave to form active caspase-1. Community-acquired methicillin-resistant *S. aureus* triggers PAMPs to induce the NLRP3 inflammasome and thus the synthesis of pro-inflammatory cytokines IL-1β and IL-18 (41). ROS production in lung endothelial cells is enhanced by polymorphonuclear neutrophil NADPH oxidase (35).

### Endothelial NOD-like Receptor Pyrin Domain 3 Inflammasome Inhibitors

As studies have shown that the NLRP3 inflammasome complex is critical for initiating inflammatory responses and plays a role in endothelial dysfunction, inhibitors that target the NLRP3 inflammasome have been overwhelmingly outperformed. For example, statins, an anti-hyperlipidaemic drug, can protect vascular endothelial cells by inhibiting the NLRP3 inflammasome and improving vascular permeability (42). This medication reduced vascular permeability in rat aortic endothelial cells by suppressing HMGB1 release in the monolayers of endothelial cells. Fenofibrate also stimulated angiogenesis in diabetic mice endothelium precursor cells by decreasing IL-1 production and deregulating NLRP3 inflammasome activity (43).

Visfatin, a multifunctional adipokine, causes in vitro endothelial dysfunction and vascular inflammation in obese and type 2 diabetes mellitus animal models through TLR4 activation. Conversely, the inflammasome assembly blockade with MCC950, a selective NLRP3 inflammasome inhibitor, prevents this activation (44). Furthermore, anakinra therapy affects the IL-1 receptor function, preventing endothelial damage. MCC950 inhibited NF-κB activity and regulated endothelial nitric oxide synthase, reducing inflammatory hyperalgesia in mice (45).

In dengue haemorrhagic fever, a study showed that its endothelial dysfunction was attenuated by dengue envelope protein domain 3 through NLRP3 inflammasome activation. Endothelial ROS generation, TNF-α and IL-1β release, and caspase-1 activation were all triggered by this protein, contributing to endothelial cell death and pyroptosis (46). However, this study showed that pyroptosis was inhibited preferentially by the NLRP3 inhibitor OLT1177 and the caspase-1 inhibitor Z-WHED-FMK. These inhibitors alleviated such metabolic burdens on endothelial mitochondria and induced and released thrombomodulin, a marker of endothelial damage.

## Conclusion

In conclusion, inflammasome activations are controlled by various signalling mechanisms in response to specific stimuli. There is significant evidence that inflammasomes and other innate immune systems, including the complement complex, play essential roles in initiating the general inflammation process. Caspase-1 maturation converts immature inflammatory cytokines to mature IL-1 and IL-18, initiating an inflammatory defensive cascade. The nature of the pro-inflammatory setting varies, ranging from invading microorganisms and their toxins (PAMPs) to those resulting from metabolic disturbances (DAMPs). Alongside endothelial cells, macrophages, smooth muscle cells, adventitial fibroblasts and mitochondria also serve as the targets and regulators of vascular inflammation and are implicated in the pathogeneses of hypertension, diabetes and atherosclerosis. Therefore, future studies are needed to unravel the complex interactions within and between cells of the vascular system that result in NLRP3 inflammasome activation.
